# Minority Resident Physicians’ Views on the Role of Race/Ethnicity in Their Training Experiences in the Workplace

**DOI:** 10.1001/jamanetworkopen.2018.2723

**Published:** 2018-09-28

**Authors:** Aba Osseo-Asare, Lilanthi Balasuriya, Stephen J. Huot, Danya Keene, David Berg, Marcella Nunez-Smith, Inginia Genao, Darin Latimore, Dowin Boatright

**Affiliations:** 1 Department of Internal Medicine, Yale School of Medicine, New Haven, Connecticut; 2 Department of Psychiatry, Yale School of Medicine, New Haven, Connecticut; 3 Department of Social and Behavioral Sciences, Yale School of Public Health, New Haven, Connecticut; 4 Department of Emergency Medicine, Yale School of Medicine, New Haven, Connecticut

## Abstract

**Importance:**

Black, Hispanic, and Native American physicians remain underrepresented in medicine despite national efforts to increase diversity in the health care workforce. Understanding the unique workplace experiences of minority physicians is essential to inform strategies to create a diverse and inclusive workforce. While prior research has explored the influence of race/ethnicity on the experiences of minority faculty and medical students, there is a paucity of literature investigating how race/ethnicity affects the training experiences of resident physicians in graduate medical education.

**Objective:**

To characterize how black, Hispanic, and Native American resident physicians experience race/ethnicity in the workplace.

**Design, Setting, and Participants:**

Semistructured, in-depth qualitative interviews of black, Hispanic, and Native American residents were performed in this qualitative study. Interviews took place at the 2017 Annual Medical Education Conference (April 12-17, 2017, in Atlanta, Georgia), sponsored by the Student National Medical Association. Interviews were conducted with 27 residents from 21 residency programs representing a diverse range of medical specialties and geographic locations.

**Main Outcomes and Measures:**

The workplace experiences of black, Hispanic, and Native American resident physicians in graduate medical education.

**Results:**

Among 27 participants, races/ethnicities were 19 (70%) black, 3 (11%) Hispanic, 1 (4%) Native American, and 4 (15%) mixed race/ethnicity; 15 (56%) were female. Participants described the following 3 major themes in their training experiences in the workplace: a daily barrage of microaggressions and bias, minority residents tasked as race/ethnicity ambassadors, and challenges negotiating professional and personal identity while seen as “other.”

**Conclusions and Relevance:**

Graduate medical education is an emotionally and physically demanding period for all physicians. Black, Hispanic, and Native American residents experience additional burdens secondary to race/ethnicity. Addressing these unique challenges related to race/ethnicity is crucial to creating a diverse and inclusive work environment.

## Key Points

**Question:**

How do minority resident physicians view the role of race/ethnicity in their training experiences?

**Findings:**

This qualitative study of 27 minority resident physicians found that participants described 3 major themes: a daily barrage of microaggressions and bias, minority residents tasked as race/ethnicity ambassadors, and challenges negotiating professional and personal identity while seen as “other.”

**Meaning:**

Results of this study suggest that minority residents face extra workplace burdens during a period already characterized by substantial stress, warranting further attention from educators, institutions, and accreditation bodies.

## Teaser

This qualitative study characterizes how black, Hispanic, and Native American resident physicians experience race/ethnicity in the workplace during their graduate medical education and training.

## Introduction

Black, Hispanic, and Native American physicians remain underrepresented in medicine despite national efforts to increase diversity in the health care workforce.^1^ Although these 3 racial/ethnic groups compose one-third of the US population, black, Hispanic, and Native American physicians constitute only 9% of practicing physicians.^2^ Moreover, the proportions of black, Hispanic, and Native American physicians have not changed substantially in the last 30 years.^2^ Exploring the role of race/ethnicity in the professional lives of minority physicians is an essential step toward identifying barriers that hinder workforce diversity and developing interventions that foster diverse work environments. Prior literature has shown that race/ethnicity has a substantial influence on underrepresented minority faculty because they are less likely to be promoted, obtain less National Institutes of Health funding, and receive lower compensation than their counterparts of white race/ethnicity.^3,4,5,6,7,8^ Research among medical students has demonstrated racial/ethnic disparities in the receipt of academic awards and in the use of positive language in letters of evaluation.^9,10^ While prior research has explored the influence of race/ethnicity on the experiences of minority faculty^3,11,12^ and medical students,^13,14^ less is known about how race/ethnicity affects the training experiences of resident physicians in graduate medical education.

Residency is a critical time during which trainees develop an understanding of how they fit into their work environment and generate plans for career development. Yet, the transition from medical resident to independent practitioner is a tenuous period for minority physicians.^15^ In that study, minority physicians were almost 30% more likely to withdraw from residency than their counterparts of white race/ethnicity and were 8 times more likely to take extended leaves of absence. Few studies have examined the factors that may underlie these disparities, although a single-site study^16^ among 19 residents of black race/ethnicity reported training experiences characterized by pervasive discrimination, lower expectations from supervisors, harsher consequences for mistakes, and social isolation. However, that study was conducted more than a decade ago, and we found no relevant follow-up research since that time.

Further investigation into the experiences of minority resident physicians is crucial to develop evidence-based best practices to enhance minority recruitment and retention. In this study, we conducted in-depth one-on-one interviews among a diverse group of minority residents to characterize the role of race/ethnicity in their workplace experiences. We used qualitative methods to identify recurrent and common themes.

## Methods

### Study Design

We performed a qualitative study of underrepresented minority resident physicians. We conducted semistructured in-depth one-on-one qualitative interviews at the 2017 Student National Medical Association Annual Medical Education Conference (April 12-17, 2017, in Atlanta, Georgia) with 27 underrepresented minority residents either in person (n = 26) or over the phone (n = 1). The single phone interview was necessary because of time constraints at the conference. Participants represented 21 different residency programs. All participants provided verbal informed consent to participate in the study and were made aware that their responses may be published anonymously. We offered participants food and beverages at a coffee shop for those interviews conducted in person. The study was approved by an institutional review board at Yale University. We performed the study using the Standards for Reporting Qualitative Research (SRQR) reporting guideline.

The lead author (A.O.-A.) and senior author (D. Boatright) conducted semistructured interviews that followed an interview guide (Box) but also gave interviewers flexibility to deviate from the guide to probe themes raised by participants. Interviews were audiotaped and professionally transcribed. The length of the interviews ranged from 13 minutes to 42 minutes (median, 24 minutes). We stopped conducting interviews when thematic saturation was reached.

Box. **Interview Guide**

1. Tell me a little bit about your experience as a resident so far.
2. What is it like to be an underrepresented minority at your program?
3. Can you recall a time when race affected your experiences as a resident? Tell me about it.
4. How, if at all, does race feature in your thought process as you go about your workday?
5. Have you ever experienced discrimination at your institution? If so, tell me about it.
6. If you have experienced discrimination, how did you perceive the level of support from coworkers or supervisors in those instances?
7. Suppose that you had 1 minute to talk to the CEO of your institution about the diversity climate at your program. What would you say?
8. What could administrators do to make the diversity climate better for you?

### Participants and Setting

Eligible participants were current resident physicians of any specialty who self-identified as black, Hispanic, and/or Native American. Our study group was a convenience sample derived from the 2017 Annual Medical Education Conference. This conference is sponsored by the Student National Medical Association, an organization devoted to minority physician development. The Annual Medical Education Conference is the largest national conference for students of color. The conference typically draws between 1200 and 1600 attendees annually, and we thought that this would be an information-rich environment to further explore minority residents’ experiences. We invited residents to participate at random via in-person recruitment. Some of the participants shared information about our study with their coresidents; we thereby obtained additional participants via snowball sampling. All invited residents agreed to participate.

### Data Analysis

We analyzed data using the constant comparative method, in which essential concepts from interview data were coded and compared over successive interviews to extract recurrent themes. Three of us (A.O.-A., L.B., and D. Boatright) coded the data. The coders were of Ghanaian American, Asian American, and African American descent, respectively. We ensured interrater reliability by coding the first several transcripts as a group and then individually coding and meeting together regularly to discuss data interpretation. We performed targeted reviews to examine the consistency of data among interviewees. We used Dedoose (https://www.dedoose.com/), an online qualitative research database, to organize the transcript data and analysis.

## Results

Interviews were conducted with 27 resident physicians from 21 residency programs representing a diverse range of medical specialties and geographic locations. Races/ethnicities were 19 (70%) black, 3 (11%) Hispanic, 1 (4%) Native American, and 4 (15%) mixed race/ethnicity; 15 (56%) were female. Table 1 lists participant characteristics. Residents represented a broad range of races/ethnicities, academic vs community practices, geographic locations, and specialties. Residents described the following 3 major themes concerning their training experiences in the workplace: (1) a daily barrage of microaggressions and bias, (2) minority residents tasked as race/ethnicity ambassadors, and (3) challenges negotiating professional and personal identity while being seen as “other.” Table 2 lists illustrative quotations representative of major themes and subthemes.

**Table 1.** Demographics of 27 Participating Minority Resident Physicians

Demographic	No. (%)
Female	15 (56)
Race/ethnicity	
Black	19 (70)
Hispanic	3 (11)
Native American	1 (4)
Mixed	4 (15)
Program type	
Academic	21 (78)
Community	4 (15)
Combined	1 (4)
Did not state	1 (4)
Geographic location	
Midwest	8 (30)
Northeast	8 (30)
West	7 (26)
South	3 (11)
Did not state	1 (4)
Year in residency training	
PGY1	5 (19)
PGY2	7 (26)
PGY3	13 (48)
PGY4	2 (7)
Specialty	
Anesthesiology	1 (4)
Emergency medicine	5 (19)
Family medicine	5 (19)
Internal medicine	1 (4)
Neurology	1 (4)
Obstetrics and gynecology	1 (4)
Pediatrics	3 (11)
Psychiatry	3 (11)
Radiology	4 (15)
Surgery	2 (7)
Urology	1 (4)

Abbreviation: PGY, postgraduate year.

**Table 2.** Themes and Subthemes, With Illustrative Quotations

Theme or Subtheme	Illustrative Quotation
**A Daily Barrage of Microaggressions and Bias**	
Alien in one’s own land	“Wow, you’ve really come a long way. You know, like, you know, being like a Mexican, that’s just…I didn’t expect somebody to be that well educated. And I said, Oh wow. Well, I did go to school, and I’ve been here for a while. And I’m actually not from Mexico, but I’m proud of my heritage. Actually, I’m fourth generation.”
Assumption of lower status	“There were instances where they would just call me ‘Nurse’ or would think that I’m everything except for a doctor.”
	“Oh yeah, I mean the one that happens the most frequently is patients thinking that I’m like transport. Not recognizing that I am their physician surgeon that’s going to be operating on them and just seeing that thought process go through their head. Or having patients that are like, ‘Oh you don’t really look like a doctor.’”
	“When you walk in and somebody’s like, ‘Hey, can you go fill my coffee?’”
Exoticization and assumptions of similarity	“There was an African American resident a year before me. We would constantly get confused. Even now…. We don’t look anything alike.”
	“There is one other black guy in the program, a guy from Cameroon. And we look nothing alike. But we have been called each other or I know for sure that I’ve been called his name before.”
Explicit bias	“There was a situation most recently where I was with one of my program directors and we went in a room to see a patient, and the father was irate, and then he went out to the hallway and one of the things he said to the nurses was that they sent the big black guy in the room to intimidate me.”
Barriers to reporting discrimination	“It wasn’t really dealt with, because we had this really long process of…you have to submit the issue to Ombuds and all this other stuff in terms of dealing with stuff like that.”
	“And that time from 6:00 am to noon is very hectic and very busy, especially when you’re on heavy services, that to stop the group and say, This is how I feel. Can we stop and talk about feelings for a moment?”
	“Oh, I’ve brought it up in the past, and it was just kind of pushed aside.”
Fear of repercussions	“They didn’t [report it] because it was actually their director who made the comment and then that director is a part of this committee. So, they didn’t make…and I don’t know that they even brought it up to the person when they made the comment…you’re trying to balance not upsetting someone too much where they feel like there’s going to be repercussions.”
**Minority Residents Tasked as Race/Ethnicity Ambassadors**	
Race/ethnicity ambassador	“…you get tapped to do various things, and some of it is stuff that you’re interested in and some of it is because they need, not necessarily a token individual, but somebody to be representative of all of the ideas of minorities because you have that insight.”
**Challenges Negotiating Professional and Personal Identity While Seen as “Other”**	
Pressure to assimilate	“Yeah, you just told someone who’s had an Afro for the last 2 years who finally got their hair flatironed once, and you’re like ‘Oh, your hair looks so professional!’”
Coping mechanisms	“I laughed it off.”
	“With a smile. I took it.”
	“You just kind of share stories and just kind of cope through things about maybe a patient who is insensitive about an individual wearing a garment, or an elderly man at the VA who had a physician of Asian descent, who he might have made a racial remark too.”
	“…you want to make sure that you’re good, and you want to make sure that you’re smart, and that you’re brilliant, and that they don’t have anything to say about you.”
Social isolation and scarce professional mentorship	“There aren’t a ton of people of color in positions of leadership or as attendings.”
	“The fact that there aren’t many of us in the program. For example, in my program, they have over 100-something residents and not even 10 are minorities.”

### “I’ve never been called ‘transport’ so many times in my life”: A Daily Barrage of Microaggressions and Bias

Participants reported regularly encountering racially/ethnically motivated behaviors from numerous sources, including coresidents, attendings, program leadership, ancillary staff, and patients. Such encounters fell on a spectrum ranging from nuanced interactions to glaring racism. While participants noted experiences of overt bias, the predominant findings were subtle exchanges that sent denigrating messages to minority residents.

Residents revealed persistent inquiries from coresidents, attendings, ancillary staff, and patients regarding their racial/ethnic background. These interactions often extended beyond polite curiosity and conveyed a message that minority residents were viewed as foreigners. One Latino resident recounted a common experience: “‘Oh, wow, that last name is different. How do I say it? Where is that from? Is English your first language? Where are you from?’ They would not just accept Texas.”

Participants also described being mistaken for nonmedical staff in the hospital by patients, patient families, and ancillary staff. This misidentification occurred despite efforts to assert belonging, including wearing white coats, stethoscopes, and identification badges and introducing themselves as physicians. As one participant noted: “…the patient’s aunt was in the room, and the next morning she told the patient’s mother that she thought I was the janitor…. She was like, ‘We’re so sorry. She told me, Oh, the janitor was so smart. He was telling everybody else what to do. He really knew his stuff.’”

Residents also noted that commonplace race/ethnicity-related features seemed to be glamorized by peers and staff members. For the participants, many of these experiences related to hair and in some cases went so far as invading personal space. As one participant explained: “There was a senior resident that spent a whole week trying to figure out what my identity and ethnicity was just by looking at my hair. One day, it was like, ‘Are you mixed with Italian? Are you mixed with Mexican?’ And then, by Friday, he actually went and grabbed my hair.”

While residents described being made to perceive themselves as exotic, they also noted receiving messages that they were indistinct from fellow resident physicians of color. Study participants were routinely mistaken for other minority residents. These experiences made residents perceive themselves as invisible at work: “Six of us are black women. They’re constantly interchanging our names, constantly interchanging people that don’t even look alike….”

Although predominant descriptions were reports of microaggressions, participants recalled encountering explicit bias from patients. Here, a Hispanic resident recounts a patient’s xenophobic monologue: “…someone like you should go back to where you came from. You’re taking advantage of our resources, and there’s all these students that would like to get into medical school that are here and from the US and don’t get in. And then you people come, and you take our places, and you take our jobs. And you don’t even have citizenship, and you don’t even speak English.”

Despite describing frequent experiences with bias, residents expressed reluctance to report events to program leadership. Multiple barriers prevented open discussions. A sense of hierarchical vulnerability precluded residents from reporting instances of bias, particularly when perpetrators were faculty. Residents worried that speaking out would incite retribution. Here, a resident describes her perceptions after her surgery attending stated that African American persons have scientifically proven thicker skin than persons of white race/ethnicity: “When you’re at certain levels of your training, you don’t have clout to really stick your neck out and say, ‘You’re totally out of line.’”

Other reasons cited for not reporting bias included beliefs that such efforts would be met with inaction, fear of being perceived as “playing the race/ethnicity card,” and difficulty expending time and mental energy. One resident said: “That’s the hottest piece of currency that I own in residency is my time. I don’t wanna spend it reliving something.”

### “The black people are asked to fix the black problems”: Minority Residents Tasked as Race/Ethnicity Ambassadors

Participants noted being expected to perform the role of race/ethnicity ambassador at their institutions as they were called on to fix problems related to diversity, shoulder additional care for minority patients, and serve as experts on racial/ethnic issues. Residents also described implementing diversity curricula at their institutions. Often, they were the only individuals working on such initiatives. One psychiatry resident stated: “The onus is on you as the minority residents or the people who care about this to take it on as an additional project.”

Despite being tasked with the responsibility to develop curricula to enhance diversity and inclusion, residents thought that their training institutions allocated insufficient resources for programming to be successful: “We have this curriculum that’s supposed to teach us about diversity, and culture, and inclusion…there’s no lunch provided…the room didn’t have enough seats for everybody. We brought that to the attention of the administration…they responded, ‘Okay, if you feel something is wrong, then you all should do something about it.’”

Minority residents also described institutions as failing to create sustainable solutions to enhance diversity and inclusion. The strategy for creating a more diverse workplace commonly relied on the efforts of a few minority faculty and residents. Because of the transitory nature of residency, minority residents expressed doubt that their initiatives would persist after they graduated. The success of diversity recruitment efforts depending on the work of minority faculty was also thought to be tenuous considering the limited number of minority faculty, whom residents perceived as having high turnover: “When we had our black attending, he was very involved in recruiting minorities, so every year there was at least one. Since he’s left…without that voice on the table, there’s few.”

While residents described experiencing a sense of pride in their diversity work, residents also acknowledged that this work demanded a sacrifice in time that would otherwise be dedicated to personal wellness or education. One resident recalled his perceptions when asked to serve on a diversity committee: “No. I do so much already. I can’t. That’s the minority tax right there…it’s great that it’s happening, but it’s just not plausible for me. I need my space. I need to hang out with my dog. I need to spend time with my girlfriend. I need to read and learn to become a better doctor. I need to do all these other things, so it’s just tiring sometimes.”

### “If you don’t have a sense of identity, then you feel very lost and you might not even finish”: Challenges Negotiating Professional and Personal Identity While Seen as “Other”

Participants described residency as a time to develop a professional identity. While acknowledging that this period is difficult for all residents, our study participants noted additional challenges secondary to race/ethnicity. These challenges included a schism between personal and work identity, pressure to assimilate, social isolation, and minimal professional mentorship.

Minority residents reported that aspects of their cultural identity were ignored at work. Residents perceived pressure to assimilate into the social culture specific to their institution while noting that their residency programs made little effort to integrate aspects of minority culture into the educational environment. These experiences intensified a sense of “otherness” among minority residents. One Hispanic resident commented: “For me, it’s like I have this part of my culture that just never gets really any attention during the workday…it’s not like I get to share any part of my culture because people don’t necessarily know anything about it to ask or seem to really care.”

Participants described the need to look outside of their work environment to find a social network to support challenges unique to their training experience. Residents expressed perceptions that aspects of their identities were in conflict and that they had to make a difficult choice between personal identity and work identity. Either choice was perceived as coming at a cost: “My significant other’s a part of a black association. Am I going to go to that or am I going to go to the meeting with the people at the hospital? What’s more important? How do I maintain both of these networks? And I had to kind of pick and balance when I’m going to do more of the academic kind of focus type of stuff as opposed to also making sure I get the community support that I feel I need.”

Minority residents also reported perceptions of being compelled to downplay, disguise, or transform their true identities to be accepted at work. These experiences often came with the implication that certain aspects of racial/ethnic identity lack professionalism. For minority residents, this perception created a duality between the piece of identity that they could display at work and their authentic selves. One resident, who wears his natural hair tied back in a ponytail, shared: “I have a pretty big ponytail…somebody came at me in a very kind of delicate, I don’t want to seem racist, way, ‘You know there’s people here who you’re going to see in clinic that probably would not feel comfortable with your hair being like that.’”

As minority residents navigated challenges to their identity, they developed multiple coping strategies. Some residents described making a conscious choice to mentally separate their authentic identity from their work environment—which they often perceived to be threatening—and adopt a stance of hypervigilance: “I get in work mode and I prepare myself. I put my shield up because you never know who’s going to be acting what kind of way in the department. I just can’t be how I am at home or with my friends.”

Residents also expressed an overwhelming pressure to be perfect to bring stability to personal identity and assert their belonging in the workplace. This behavior led to a heightened awareness about the perception of external factors, including speech patterns, body language, and clothing: “You just want to make sure that what you’re doing is top-notch, because you know for others, they may use your mistakes and then kind of pair that with your race.”

In many cases, challenges of personal identity were exacerbated by limited social and professional support. As minority residents attempted to find ways to be their true selves in the workplace, race/ethnicity was thought to be a barrier to fulfilling mentoring relationships, reinforcing perceptions of isolation. While minority faculty mentors were generally desired by our study population, they were in short supply.

## Discussion

Participants in our study described 3 recurring scenarios in residency training. First, minority residents routinely experience racial/ethnic bias at work and are reluctant to report it to their programs. Second, residency programs lack institutionalized systems to promote diversity and rely on minority residents to fulfill these tasks. Third, minority residents encounter challenges balancing professional and personal identity.

Instances of bias previously described in the literature have often been explicit.^11,13,14,17,18^ Results from our study suggest that the bias encountered by minority resident physicians is increasingly multidimensional and covert. This distinction is important because subtle manifestations of bias will likely necessitate alternative interventions.

While minority tax, or the extra burdens placed on minorities to promote diversity in their institutions, has been well characterized among faculty,^12,19,20^ our results indicate that minority physicians may experience these earlier in their career than previously reported, during a time already characterized by substantial educational demands and mental stress. These experiences may limit educational opportunities for minority residents and contribute to burnout.

Minority residents reported perceiving themselves as outsiders at work. While social isolation and inadequate mentorship have been described in prior studies^12,20^ of minority physicians, residents in our study further described challenges reconciling their professional and personal identity and often perceived pressure to conceal aspects of their racial/ethnic identity. These findings are important because identity suppression is associated with lower job satisfaction and higher turnover among targeted individuals.^21^

### Limitations

Our study has several limitations. Participants were recruited at a conference dedicated to minority physician advancement. Residents who were present may be more attuned to issues pertaining to diversity and inclusion. However, residents who attended this conference were sponsored by institutions that supported such outreach efforts, and these institutions may have climates friendlier to minority residents. In addition, most participants were of black race/ethnicity, and our data may not capture the full experience of other minority groups. As is inherent to all qualitative work, our research is meant to be hypothesis generating. Themes that emerged should be tested in future quantitative work to further explore their prevalence and influence on the careers of minority physicians and workforce diversity.

### Implications

Results of our study have important policy implications for residency training institutions, residency program leadership, and the Accreditation Council for Graduate Medical Education (ACGME). The frequent instances of bias reported by our study participants should be of interest to residency program directors. Program leadership should note that workplace bias is likely underrecognized by institutions. Unfavorable treatment on the basis of race/ethnicity has been associated with higher physician turnover and lower job satisfaction.^17^ Consequently, residency program directors should be proactive about developing formal methods to monitor and address instances of bias experienced by residents.

The burden of minority residents to promote diversity is one that can be minimized. Diversity efforts should not be the function of trainees, whose principal responsibility is the pursuit of clinical excellence. For residents who choose to lead diversity initiatives, residency programs should prioritize resource allocation, including administrative support and institutional funding. Residency program leadership ought to regard challenges related to diversity and inclusion as an institutional problem and strive to achieve institutional excellence.

Minority physicians reported difficulty being their “true selves” at work and faced challenges reconciling their professional and personal identity. As the workplace becomes increasingly diverse, residency program leadership should encourage trainees to bring their “whole selves” to work. Organizations that fail to allow whole selves in the workplace limit their access to crucial sources of creativity and innovation within individuals.^22^ To promote bringing whole selves to work, programs would do well to create a more robust support network for minority residents. Efforts to support minority residents can include creating open forums to discuss race/ethnicity-related issues as a way to process experiences of bias. Another strategy includes efforts to increase the number of minority faculty. Residency programs with few minority faculty should aggressively recruit new minority staff. Increasing the number of minority faculty will provide tangible mentorship to minority residents, enhance the diversity in the learning environment, and potentially reduce implicit bias in the workplace.^23^

The institutional abdication of responsibility by residency programs to promote the efforts of diversity and inclusion may be of interest to the ACGME. Recently, the ACGME updated their Common Program Requirements to reflect an expectation that all residency programs promote recruitment and retention of a diverse workforce.^24^ This requirement will go into effect in July 2019, and residency programs will have 1 year to adjust their practices before citations will be implemented.^24^ This approach is in line with similar diversity accreditation standards at the undergraduate medical education level,^25^ which have been in place since 2009.

While the ACGME has taken an important first step, there is much work to be done to fully operationalize this statement and provide guidance to residency programs. Despite medical school diversity standards being in place for almost a decade, medical schools struggle to adhere to these standards.^26^ Between 2011 and 2014, a total of 27% of medical schools were not in compliance with diversity standards, and an additional 29% were in need of monitoring.^27^ To avoid a similar trend among residency programs, the ACGME should create benchmarks for excellence, such as bias education programming and formalized diversity committees. Moreover, the current ACGME position statement makes no mention of the experience of minority residents.^28^ While recruitment and retention are key components of the diversity landscape, our findings indicate that more work is needed to optimize the experiences of minority residents. Focusing on obtaining higher numbers of minorities without addressing the specific challenges that minority residents encounter in the workplace is likely to result in continued disparities.

## Conclusions

Results of our study suggest that minority resident physicians face extra workplace burdens during a period already characterized by substantial stress. Addressing these racial/ethnic challenges is crucial to creating a diverse and inclusive workforce.
